# Supplementary material for: Mate choice in fruit flies is rational and adaptive
Source: Nat Commun. 2017 Jan 17;8:13953. doi: 10.1038/ncomms13953 (PMC5247575; doi:10.1038/ncomms13953)
Supplement: Supplementary Information — Supplementary Figures [file ncomms13953-s1.pdf]

Supplemental Figures

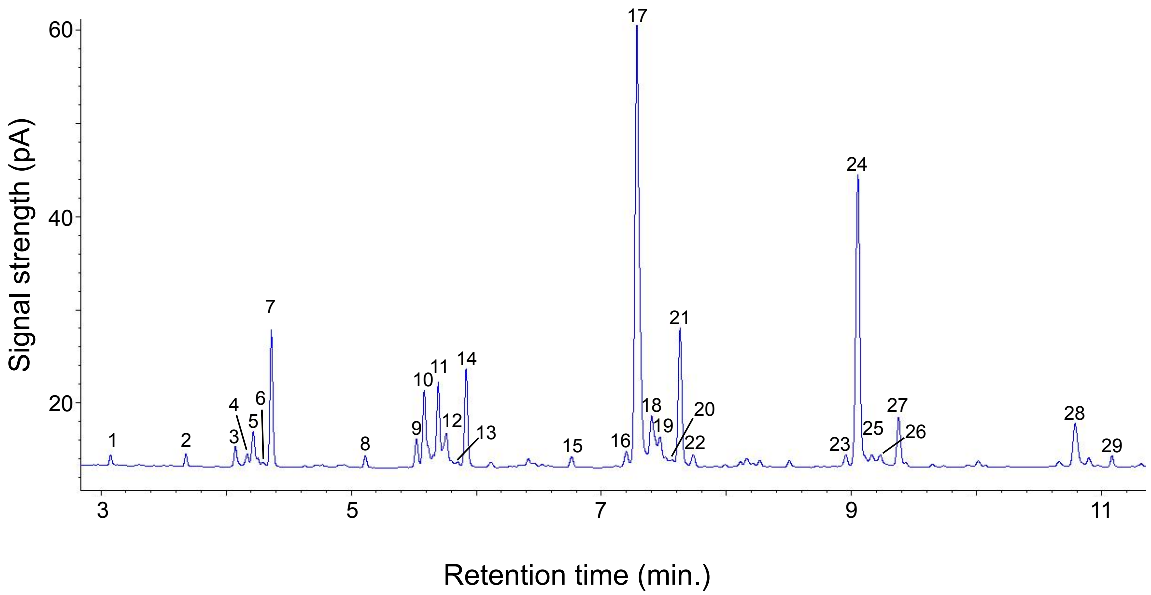

Supplementary Figure 1. Female cuticular hydrocarbon (CHC) profile. We integrated CHCs in the order of retention times during gas chromatography, representing all CHCs that could be reliably integrated for all sampled females.

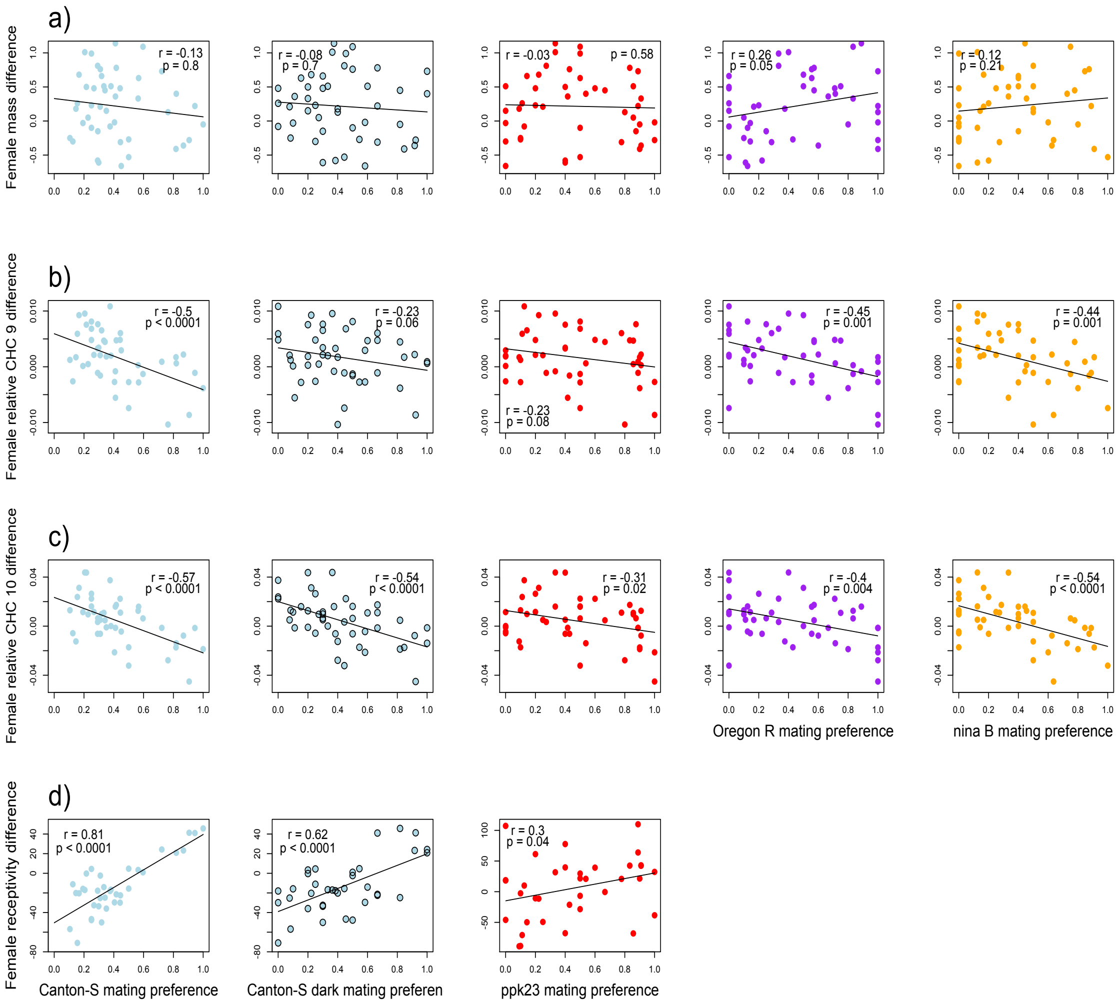

Supplementary Figure 2. Relationships between male mate choice and female traits. Correlations between the proportions of the first female line mated for all pairwise combinations for each male tested and pairwise differences in (a) female mass, (b) relative abundance of CHC 9, (c) relative abundance of CHC 10, and (d) female receptivity. Correlations and significance were determined using permutation tests to avoid pseudoreplication. Canton-S mating preferences represent the average mating bias across two independent blocks measuring mating preference. Regression lines for all plots represent geometric mean regression.
